# Angiomotin regulates prostate cancer cell proliferation by signaling through the Hippo-YAP pathway

**DOI:** 10.18632/oncotarget.14358

**Published:** 2016-12-29

**Authors:** Hao Zeng, Angelica Ortiz, Peng-Fei Shen, Chien-Jui Cheng, Yu-Chen Lee, Guoyu Yu, Song-Chang Lin, Chad J. Creighton, Li-Yuan Yu-Lee, Sue-Hwa Lin

**Affiliations:** ^1^ Department of Urology, Institute of Urology, West China Hospital, Sichuan University, Chengdu, China; ^2^ Department of Translational Molecular Pathology, The University of Texas M. D. Anderson Cancer Center, Houston, Texas, USA; ^3^ The University of Texas Graduate School of Biomedical Sciences at Houston, Texas, USA; ^4^ Department of Pathology, School of Medicine, College of Medicine, Taipei Medical University, Taipei, Taiwan; ^5^ Department of Pathology, Taipei Medical University Hospital, Taipei, Taiwan; ^6^ Dan L. Duncan Cancer Center, Baylor College of Medicine, Houston, Texas, USA; ^7^ Department of Medicine, Baylor College of Medicine, Houston, Texas, USA; ^8^ Department of Genitourinary Medical Oncology, The University of Texas M. D. Anderson Cancer Center, Houston, Texas, USA

**Keywords:** angiomotin, Hippo pathway, YAP, BMP4, proliferation

## Abstract

Angiomotin (AMOT) is a family of proteins found to be a component of the apical junctional complex of vertebrate epithelial cells and is recently found to play important roles in neurofibromatosis type 2 (NF-2). Whether AMOT plays a role in prostate cancer (PCa) is unknown. AMOT is expressed as two isoforms, AMOTp80 and AMOTp130, which has a 409 aa N-terminal domain that is absent in AMOTp80. Both AMOTp80 and AMOTp130 are expressed in LNCaP and C4-2B4, but at a low to undetectable level in PC3, DU145, and BPH1 cells. Further study showed that AMOTp130 and AMOTp80 have distinct functions in PCa cells. We found that AMOTp80, but not AMOT p130, functioned as a tumor promoter by enhancing PCa cell proliferation. Mechanistic studies showed that AMOTp80 signaled through the Hippo pathway by promoting nuclear translocation of YAP, resulting in an increased expression of YAP target protein BMP4. Moreover, inhibition of BMP receptor activity by LDN-193189 abrogates AMOTp80-mediated cell proliferation. Together, this study reveals a novel mechanism whereby the AMOTp80-Merlin-MST1-LATS-YAP-BMP4 pathway leads to AMOTp80-induced tumor cell proliferation.

## INTRODUCTION

Angiomotin (AMOT) is a family of proteins originally identified as an angiostatin binding protein that regulates endothelial cell migration and tube formation [1, 2]. AMOT is subsequently found to be a component of the apical junctional complex of vertebrate epithelial cells [3, 4]. The role of AMOT in disease has not been extensively investigated. AMOT was recently found to play important roles in neurofibromatosis type 2 (NF2), breast cancer, and renal cell carcinoma [5–7]. Whether AMOT plays a role in prostate cancer (PCa) progression and how AMOT signals in PCa are still unknown.

AMOT has been shown to regulate many cellular functions by serving as an adaptor protein in different cellular contexts. In epithelial cells, AMOT could interact with the cell junction protein RICH1, and this interaction leads to the localization of AMOT to the apical membrane of polarized epithelial cells [3, 4]. In NF2, loss of AMOT interaction with NF2/Merlin could increase its interaction with RICH1, leading to Rac activation [5]. In breast cancer, AMOT was proposed to coordinate the dysregulation of cell polarity with the induction of neoplastic growth [6]. In endothelial cells, AMOT was shown to regulate endothelial cell migration and tube formation [1, 2]. AMOT-mediated cell migration was also critical in embryogenesis as knockout of AMOT in zebrafish and in mouse led to embryonic lethality due to defects in cell migration into proximal extra-embryonic regions [8, 9].

AMOT family includes AMOT, AMOT-L1, and AMOT-L2; and AMOT is expressed as two different isoforms, AMOTp80 and AMOTp130, with the latter containing a 409 aa N-terminal domain that is absent in AMOTp80 [10]. Several studies showed that AMOTp130 and AMOTp80 have distinct functions. In endothelial cells, AMOTp80 isoform could enhance cell migration, while AMOTp130 isoform was associated with actin and can affect cell shape [2, 11]. This is likely due to the N-terminal domain of AMOTp130 being able to interact with actin and the tight junction associated protein MAGI-1 [12–14]. AMOTp80 has been shown to play a role as a tumor promoter, as its expression could enhance endothelial invasion and stabilize established tubes [15]. In breast cancer cells, overexpression of AMOTp80 was found to induce cell proliferation through the activation of the ERK signal pathway [6]. In contrast, AMOTp130 inhibits cell growth [16].

Recently, AMOTp130 has been shown to interact with YAP, a transcriptional coactivator downstream of the Hippo pathway [16, 17]. Hippo pathway plays a critical role in organ size control in Drosophila and is involved in tumorigenesis in mammalian cells [18]. The current model of the mammalian Hippo pathway indicates that the mammalian homolog of Hippo, Mst1/2 kinase, phosphorylates and activates the Lats1/2 kinase, which phosphorylates and inactivates YAP/TAZ. Thus, YAP/TAZ are the key components of the Hippo pathway and have been shown to play a role in tumorigenesis [19, 20]. AMOT family proteins have been shown to regulate Hippo pathway, in which the PPXY domain in the N-terminus of AMOTp130, AMOT-L1, or AMOT-L2 could interact with the WW motif of YAP or TAZ, resulting in retention of YAP or TAZ in the cytoplasm [17, 21–23]. Recently, Adler and his colleagues reported that AMOTp130 could reduce YAP stability by binding and activating the ubiquitin ligase Atrophin-1 Interacting Protein 4 (AIP4), providing a different mode of AMOTp130 interaction with the Hippo pathway [16]. Because AMOTp80 does not contain the PPXY domain, it cannot interact with YAP or TAZ directly as AMOTp130. However, evidence also showed that AMOTp80 could interact with Merlin, a tumor suppressor protein involved in Neurofibromatosis type II, to regulate mitogenic signaling and tumor suppression [5]. Interestingly, Merlin is one of the upstream regulators of the Hippo pathway and recently has been shown to regulate human meningioma cell growth by signaling through YAP [18, 24–27]. Whether AMOTp80 could regulate cellular function through the Hippo pathway is also unclear.

In this study, we determined the roles of AMOTp80 and AMOTp130 in PCa progression and found that AMOTp80 could function as a tumor promoter by enhancing PCa cell proliferation. We further showed that AMOTp80 could signal through the Hippo pathway by promoting the nuclear translocation of YAP to increase YAP target protein expression.

## RESULTS

### Expression of AMOT isoforms in PCa cell lines

**Figure 1:**
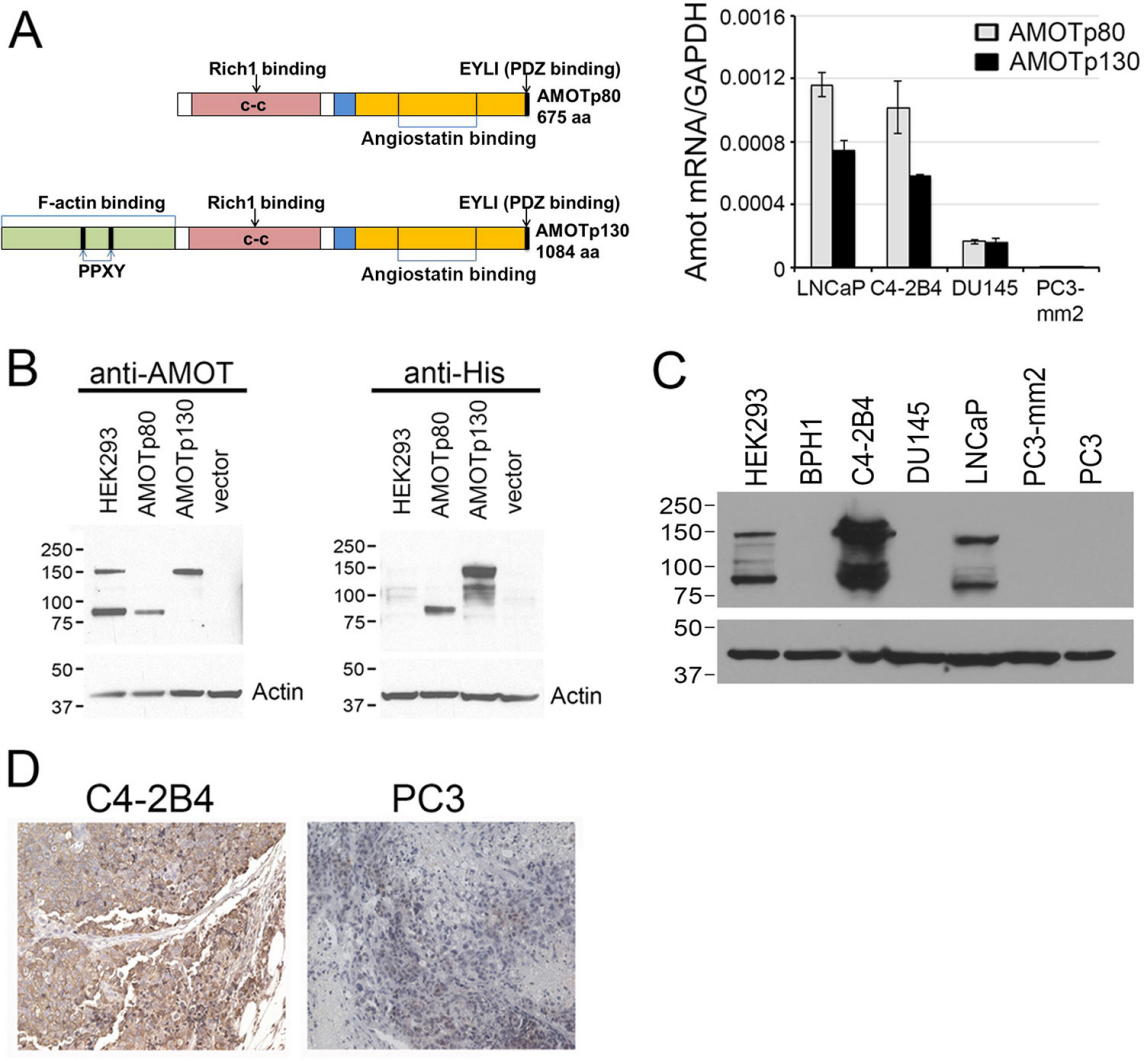
Expression of AMOT isoforms in PCa cell lines **A.** Left panel, Diagram of AMOTp80 and AMOTp130 proteins. Right panel, AMOTp80 and AMOTp130 messages were detected by real-time PCR using isoform-specific primers. **B.** Specificity of anti-AMOT antibody. PC3-mm2 cells stably expressing 7 histidine-tagged AMOTp80, AMOTp130, or vector alone were immunoblotted with anti-AMOT or anti-His antibody. HEK293 cells were used as a control for endogenous AMOT expression. **C.** Expression of AMOT isoforms in PCa cell lines. Equal amounts of cell lysates from PCa cell lines were immunoblotted with anti-AMOT antibody. Actin was used as a loading control. **D.** Subcutaneous tumors were generated from C4-2B4 and PC3 PCa cells. Formalin-fixed paraffin-embedded PCa tumors were immunostained with anti-AMOT antibody and the signals detected by DAB staining.

To examine the expression of AMOT in PCa cell lines, real-time PCR were first used to determine its message levels in these cell lines. Oligonucleotide primers specific for AMOTp80 or AMOTp130 were used to perform real-time PCR on RNAs prepared from several PCa cell lines. Among the four PCa cell lines, LNCaP and C4-2B4 expressed higher levels of both AMOTp80 and AMOTp130 messages, while AMOT messages were relatively lower or undetectable in PC3-mm2 and DU145 cells. In addition, AMOTp80 and AMOTp130 were co-expressed in C4-2B4 and LNCaP cells with similar levels. (Figure 1A)

**Figure 2:**
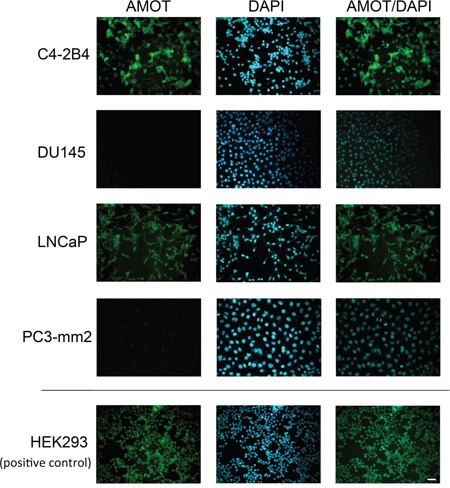
Expression of Amot in PCa cell lines PCa cells on cover slips were immunostained with anti-AMOT antibody and counterstained with DAPI. HEK293 cells were used as a positive control. Scale bar, 50 μm.

Next, the levels of AMOT proteins in these cell lines were examined. Because the commercially available anti-AMOT antibodies were not sensitive enough in detecting AMOT proteins in cell lysates or in immunohistochemistry studies, we generated polyclonal antibodies against the full-length recombinant AMOT protein. To test for the specificity of the anti-AMOT antibodies, PC3-mm2 cells were stably transfected with 7 histidine-tagged AMOTp80 or AMOTp130 cDNA. The exogenously expressed AMOTp80 and AMOTp130 were detected by anti-his antibody (Figure 1B, right panel). Affinity-purified anti-AMOT antibodies could recognize exogenously expressed as well as the endogenous AMOTp80 and AMOTp130 in HEK293T cells (Figure 1B, left panel). Using this anti-AMOT antibody, western blot of cell lysates from PCa cell lines showed that higher levels of AMOTp80 and AMOTp130 proteins were expressed in C4-2B4 and LNCaP cells than in BPH1, DU145, PC3, and PC3-mm2 cells (Figure 1C). We noted that PC3-mm2 did express a low level of AMOTp80 or AMOTp130, which could be detected upon longer exposure (data not shown). The AMOT message expression in PCa cell lines is in general agreement with the AMOT protein expression (Figure 1A and 1C). To test whether anti-AMOT antibodies could detect AMOT in immunohistochemical analyses, we generated subcutaneous tumors from C4-2B4 and PC3-mm2 cells. Immunohistochemical staining of formalin-fixed paraffin-embedded tumor sections showed moderate intensity of AMOT staining in C4-2B4 tumor, while the staining in PC3-mm2 tumor was very weak (Figure 1D). These results were consistent with those observed in western blot (Figure 1C) and immunofluorescence analyses (Figure 2). Together, these results indicate that both AMOTp80 and AMOTp130 are expressed, albeit in different levels, in PCa cell lines.

### Effect of AMOTp80 on the proliferation and migration of PCa cells

**Figure 3:**
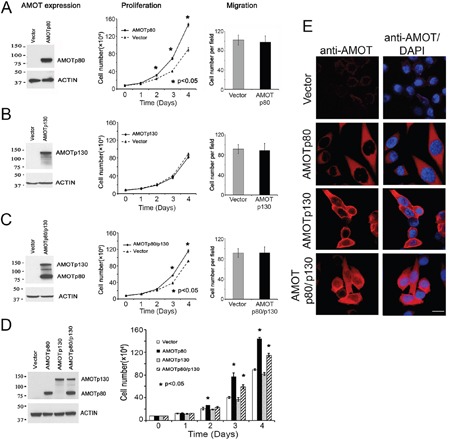
Effect of AMOT on the proliferation and migration of PC3-mm2 cells PC3-mm2 cells overexpressed **A.** AMOTp80, **B.** AMOTp130 or **C.** AMOTp80/p130 were analyzed for protein expression (left) by using anti-AMOT antibody, proliferation (middle) by using cell count, and migration (right) by using Boyden chamber assay. **D.** Proliferation of PC3-mm2 cells overexpressing AMOTp80, AMOTp130, or both was performed in the same experiment for a direct comparison. **E.** Overexpression of AMOTp80, AMOTp130, and both AMOTp80/p130 was analyzed by immunofluorescence. Scale bar, 20 μm

To examine the function of AMOTp80 in PCa cells, PC3-mm2 cells were transduced with a bicistronic retrovirus containing neomycin resistance marker and AMOTp80 cDNA. Cells expressed AMOTp80 were selected by G418 and the expression of AMOTp80 was confirmed by western blot (Figure 3A, left). Overexpression of AMOTp80 in PC3-mm2 increased cell proliferation compared to vector-transduced control cells (Figure 3A, middle). The effect of AMOTp80 on PCa migration was examined by using a Boyden Chamber migration assay. Expression of AMOTp80 did not affect the migration of PC3-mm2 cells (Figure 3A, right). However, PC3-mm2 is already highly migratory and the migration may not be further increased with expression of AMOT. These results indicate that AMOTp80 plays a role in PCa cell proliferation but not cell migration.

### Effect of AMOTp130 on the proliferation and migration of PCa cells

The role of AMOTp130 in PCa was examined by overexpressing AMOTp130 in PC3-mm2. PC3-mm2 cells were transduced with a bicistronic retrovirus containing GFP and AMOTp130 cDNA. Cells expressed AMOTp130 were selected by FACS for GFP. The expression of AMOTp130 was confirmed by western blot (Figure 3B, left). In contrast to AMOTp80, overexpression of AMOTp130 in PC3-mm2 cells did not affect either cell proliferation (Figure 3B, middle) or migration (Figure 3B, right).

### Effect of AMOTp80/p130 co-expression on PC3-mm2 proliferation and migration

As AMOTp80 and AMOTp130 are always co-expressed in PCa cells, we examined the effect of their co-expression on PC-3mm2 cell proliferation. Bicistronic retroviral vectors with neomycin or GFP selection markers were used to express AMOTp80 and AMOTp130, respectively, and cells were selected by neomycin-resistance and GFP expression. Western blot showed that both AMOTp80 and AMOTp130 were expressed (Figure 3C, left). PC3-mm2-AMOTp80/p130 cells showed an increase in proliferation compared to that in control cells transfected with both neomycin-resistance and GFP vectors (Figure 3C, middle). Co-expression of AMOTp80/p130 in PC3-mm2 cells did not have a significant effect on cell migration (Figure 3C, right).

Because overexpression of AMOTp80/p130 showed a reduced level of cell proliferation when compared to that in AMOTp80 overexpression alone (Figure 3A and 3C), we examined the effect of AMOTp80, AMOTp130, and AMOTp80/p130 on PC3-mm2 cell proliferation in the same experiment. The expression of AMOTp80, AMOTp130, and co-expression of AMOTp80/p130 were confirmed by western blot (Figure 3D, left) and immunofluorescence (Figure 3E). As shown in Figure 3D, overexpression of AMOTp80 or both AMOTp80/p130 led to significant increases in PC3-mm2 proliferation at days 3 and 4 as compared to vector control. However, the effect of AMOTp80/p130 on PC3-mm2 proliferation was less than that observed with AMOTp80 alone (Figure 3D). These observations suggest that the expression of AMOTp130 seems to reduce the effect of AMOTp80 in promoting cell proliferation.

### Effect of AMOT knockdown on C4-2B4 cells

**Figure 4:**
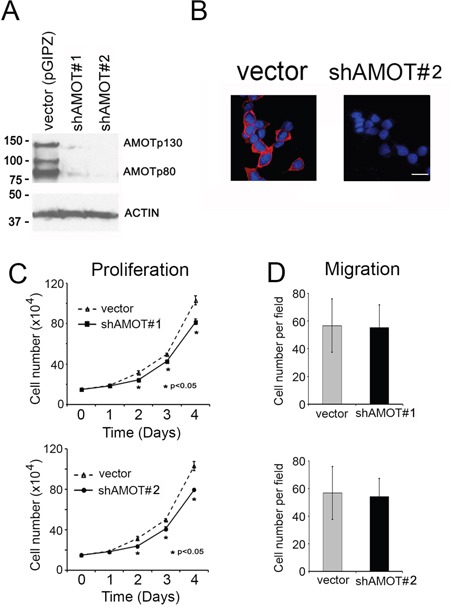
Effect of AMOT knockdown on the proliferation and migration of C4-2B4 cells **A.** Western blot of cell lysates from C4-2B4 cells transduced with pGIPZ control vector, shAMOT RNA #1 or #2 vectors using anti-AMOT antibody. **B.** Knock down of AMOT by using shRNA#2 was analyzed by immunofluorescence. Scale bar, 20 μm **C.** Proliferation of C4-2B4 cells with AMOT knockdown. **D.** Migration of C4-2B4 cells with AMOT knockdown.

Next, shRNA was used to knockdown AMOT in C4-2B4 cells that express a high level of AMOT. The effect of two AMOT shRNAs (shRNA#1 and shRNA#2), which recognize sequences shared between AMOTp80 and AMOTp130, on AMOT depletion was examined. Both shRNAs could effectively knock down AMOTp80 and AMOTp130 compared to vector-transfected C4-2B4 cells (Figure 4A and 4B). The results showed that knockdown of AMOT in C4-2B4 cells could decrease cell proliferation (Figure 4C), but not migration (Figure 4D).

### AMOTp80 inhibits Hippo pathway

One possible mechanism by which AMOTp80 could increase cell proliferation might be through the inhibition of the Hippo tumor suppressor pathway [28]. AMOTp80 has previously shown to interact with tumor suppressor NF2/merlin [5, 28]. NF2/merlin regulates mammalian tissue homeostasis through inhibiting the activity of the YAP oncoprotein, a key player of the Hippo pathway. It is possible that AMOTp80/merlin interaction might lead to an increase of YAP transcriptional activity that could increase cell proliferation. Interestingly, AMOTp130, AMOT-L1 or AMOT-L2, but not AMOTp80, have been found to increase YAP phosphorylation that increases YAP degradation or YAP cytoplasmic retention, leading to cell growth suppression [17, 21, 23].

**Figure 5:**
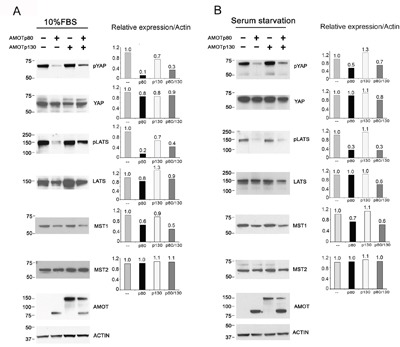
Effect of AMOT on the Hippo-YAP signaling pathway in PC3-mm2 cells **A.** Left panel, PC3-mm2 cells overexpressing AMOTp80, AMOTp130, or both AMOTp80/p130 were cultured under normal serum condition. The levels of pYAP, YAP, pLATS, LATS, MST1 and MST2 were examined by western blot. Right panel, the relative expression of these proteins after normalization against actin. **B.** To examine the influence of serum, PC3-mm2 cells overexpressing AMOTp80, AMOTp130, or both AMOTp80/p130 were cultured overnight under serum starvation condition followed with serum stimulation. Western blot was performed and normalized as described in (A).

To determine whether AMOTp80 could increase PCa cell proliferation through the inhibition of Hippo pathway, we examined the effect of AMOTp80 on the phosphorylation of YAP and LATS (an upstream protein of YAP). As shown in Figure 5A, overexpression of AMOTp80 or both AMOTp80/p130 in PC3-mm2 cells could decrease the levels of pYAP and pLATS, but not the levels of total YAP or LATS proteins under normal condition. Upstream of LATS is MST. As shown in Figure 5A, the levels of MST1, but not MST2, were similarly decreased by AMOTp80 or both AMOTp80/p130. Thus, overexpression of AMOTp80 decreases MST1-pLATS-pYAP levels in the Hippo pathway. As signal transduction that mediates growth response might be sensitive to growth stimulation from serum, we also examined the effect of AMOTp80 on the phosphorylation of YAP and LATS under overnight serum starvation followed with serum stimulation condition. As shown in Figure 5B, the changes of pYAP and pLATS induced by overexpression of AMPTp80 were similar to those in normal serum condition. Thus, the effect of AMOTp80 on pYAP and pLATS is likely independent of serum stimulation.

**Figure 6:**
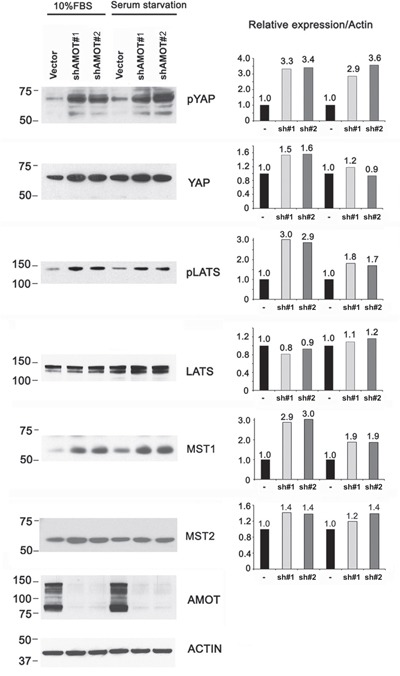
Effect of AMOT on the Hippo-YAP signaling pathway in C4-2B4 cells C4-2B4 cells were transduced with pGIPZ control vector, AMOT shRNA #1, or AMOT shRNA#2 in pGIPZ vectors under normal serum condition or serum starvation overnight followed with serum stimulation condition. Western blot was used to detect the expression levels of the proteins and changes in their phosphorylation after normalization against actin.

We also examined whether knockdown of AMOTp80/p130 in C4-2B4 cells could affect the phosphorylation of YAP and LATS. As shown in Figure 6, significant increases in the levels of pYAP, pLATS, and MST1 were observed in C4-2B4 cells with AMOTp80/p130 knockdown. Similar to those observed in PC3-mm2 cells, the effects of AMOTp80/p130 on pYAP and pLATS were also not affected by serum starvation (Figure 6). Together, these observations suggest that AMOTp80 expression inhibits the Hippo pathway and the effect of AMOTp80 on Hippo pathway is independent of stimulation from serum growth factors.

### AMOTp80 increases the nuclear localization of YAP

**Figure 7:**
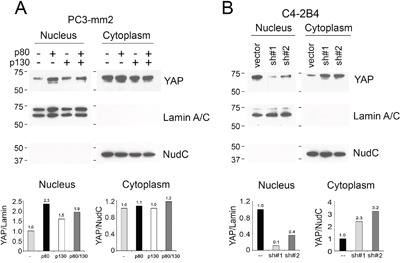
Effect of AMOT on the cellular localization of YAP Nuclear and cytoplasmic fractionation of **A.** PC3-mm2 cells overexpressing AMOTp80, AMOTp130, or both; or **B.** C4-2B4 cells with AMOT knockdown. The relative levels of YAP in the nucleus and cytoplasm were normalized to Lamin A/C, a protein found in the nucleus, or NudC, a protein found in the cytoplasm, respectively.

The Hippo pathway can inhibit the transcriptional activity of YAP by cytoplasmic retention or degradation of YAP in a phosphorylation-dependent manner [17, 22, 23]. Because overexpression of AMOTp80 in PC3-mm2 cells decreases the levels of pYAP (Figure 5A) and knockdown of AMOTp80/p130 in C4-2B4 cells increases the phosphorylation of YAP and LATS (Figure 6), we examined whether overexpression of AMOTp80 could affect YAP nuclear localization. Nuclear/cytoplasmic fractionation showed that overexpression of AMOTp80 in PC3-mm2 cells led to a higher level of YAP protein in the nuclear fraction compared to that in control cells (Figure 7A). Overexpression of AMOTp130 in PC3-mm2 cells did not have a significant effect on nuclear localization of YAP (Figure 7A). Overexpression of both AMOTp80 and AMOTp130 showed an increase of nuclear YAP, however, the level of nuclear YAP was less than that with AMOTp80 alone (Figure 7A). The up-regulation of nuclear YAP mirrored the down-regulation of pYAP in PC3-AMOTp80 cells (Figure 5A, 5B). In AMOT knockdown C4-2B4 cells, nuclear localization of YAP decreased accompanied with an increase of YAP in the cytoplasmic fraction compared to that in vector-transfected C4-2B4 cells (Figure 7B). The down-regulation of nuclear YAP in AMOT knockdown C4-2B4 cells mirrored the upregulation of pYAP (Figure 6). These observations suggest that overexpression of AMOTp80 could increase nuclear translocation of YAP, likely due to decreases in the levels of pYAP.

It is of interest to compare the levels of YAP in PC3-mm2 and C4-2B4 cells. In the studies shown in Figure 7, we used the same cell number for PC3-mm2 (see non-transfected control lane) and C4-2B4 cells (see vector-transfected lane) and the exposure time was similar. Thus, it seems that the total levels (cytoplasmic plus nuclear) of YAP in PC3-mm2 and C4-2B4 cells are similar. However, the level of nuclear YAP in C4-2B4 is higher than that in PC3-mm2, probably due to the endogenous expression of AMOTp80 in C4-2B4 cells.

### AMOTp80 increases YAP target gene expression

**Figure 8:**
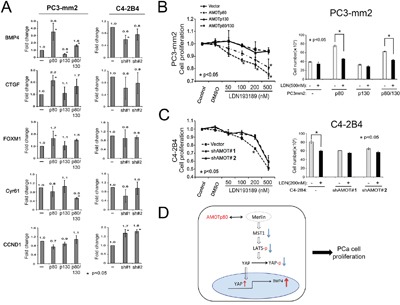
Effect of AMOT on YAP target gene expression and the involvement of BMP4 in AMOT-mediated PCa cell proliferation **A.** PC-3mm2 cells overexpressing AMOTp80, AMOTp130, or both, and C4-2B4 cells with knock-down of AMOT by pGIPZ control vector, shAMOT RNA #1 or #2 in pGIPZ vectors. YAP target genes were detected by real-time PCR using specific primers. **B.** Left panel, PC3-mm2 cells overexpressing AMOTp80, p130, or both were incubated with an increasing concentration of LDN193189, a BMP Type I receptor antagonist, for two days and the number of cells was counted. Right panel, PC3-mm2 cells overexpressing AMOTp80, p130, or both were treated with 500 nM LDN193189. **C.** Left panel, C4-2B4 cells with AMOT knockdown were treated with an increasing concentration of LDN193189 and the number of cells was counted. Right panel, C4-2B4 cells with AMOT knockdown were treated with 200 nM LDN193189. **D.** Working model of AMOTp80-mediated PCa cell proliferation. AMOTp80 likely interacts with Merlin and this leads to decreases in the levels of MST1, pLATS and pYAP. The ensuing decrease in YAP phosphorylation leads to an increase in the nuclear translocation of YAP. YAP activation stimulates the transcription of BMP4, resulting in an increase in PCa cell proliferation.

YAP is a transcription coactivator [29, 30]. Proliferation related target genes of YAP include cell cycle related genes (CCND1, FOXM1), connective tissue family member (CTGF, Cyr61), and bone morphogenetic protein 4 (BMP4) [30, 31]. We examined whether the expression of these YAP target genes is affected by AMOTp80 overexpression using real-time PCR. As shown in Figure 8A, BMP4 was significantly upregulated by AMOTp80 overexpression in PC-3mm2 cells, while CTGF, FOXM1 showed moderate increases and Cyr61 and CCND1 did not show significant changes. Moreover, BMP4 expression was reduced in C4-2B4 cells with AMOTp80 knockdown compared to vector control. These observations suggest that BMP4 is one of the downstream target genes of the AMOTp80-YAP pathway in PCa cells.

### Inhibition of BMP4 receptor signaling inhibits AMOTp80-induced cell proliferation

Finally, we examined whether BMP4 was part of the signaling pathway of AMOTp80-induced cell proliferation. PC3-mm2 cells overexpressing AMOTp80 or p130 or both and C4-2B4 cells with AMOTp80 knockdown were treated with an increasing concentration of LDN193189, an inhibitor of the BMP Type I receptors, for two days and the number of cells was counted. PC3-mm2 cells overexpressing AMOTp80 or both p80/p130 showed a concentration-dependent inhibition by LDN193189 (p<0.05) on cell proliferation (Figure 8B, left panel), whereas the vector control or PC3-mm2 overexpressing AMOTp130 cells did not show significant inhibition (Figure 8B, left panel). The effect of LDN193189 at the concentration of 500 nM on the proliferation of PC3-mm2 AMOT overexpressing cells was further examined and LDN193189 was found to consistently inhibit the proliferation of PC3-mm2/AMOTp80 and PC3-mm2/AMOTp80/p130, but not the control and PC3-mm2/AMOTp130 cells (Figure 8B, right panel). Similarly, treatment of C4-2B4/Vector cells with LDN193189 led to a concentration-dependent inhibition up to 200 nM, while C4-2B4/shAMOT#1 or #2 cells were not responsive. Treatment with 500 nM LDN193189 showed an inhibition of both control C4-2B4/Vector and AMOT knockdown cells, likely due to toxicity (Figure 8C, left panel). Together, these results suggest that inhibition of BMP4 via BMP receptor signaling blocks the AMOTp80-mediated proliferation of PCa cells.

Collectively, our studies suggest that the pathway for AMOTp80-mediated PCa cell proliferation is follows. AMOTp80 likely interacts with Merlin and this interaction leads to a decrease in the levels of MST1, pLATS and pYAP. The ensuing decrease in YAP phosphorylation leads to an increase in the nuclear translocation of YAP. YAP activation stimulates the transcription of BMP4, resulting in an increase in PCa cell proliferation (Figure 8D).

## DISCUSSION

We have shown that AMOTp80, but not AMOTp130, plays a role in enhancing PCa cell proliferation. In addition, we showed that one of the mechanisms of AMOTp80-mediated proliferation is through inhibition of the Hippo pathway, resulting in nuclear translocation of YAP and an increased expression of the YAP target gene BMP4. We further showed that AMOTp80-mediated BMP4 expression promotes PCa cell proliferation. Together, our studies identify AMOTp80-MST1-LATS-YAP-BMP4 as a novel pathway that plays a role in PCa progression.

Isoforms of AMOT have distinct functions in cellular processes. AMOTp130 has been shown to associate with cytoskeleton actin filaments and is involved in vessel stabilization and maturation during angiogenesis [11, 13]. In recent years, AMOTp130 was discovered as a tumor suppressor through activation of the Hippo pathway [16, 17, 21–23]. In contrast, AMOTp80 has been considered to promote the migration of endothelial cell in the angiogenic process [13]. Interestingly, Ernkvist et al. [13] found that when both isoforms of AMOT were expressed in aortic endothelial cells, the ability of AMOTp80 to promote cell motility was dominant over the inhibitory effect of AMOTp130.Moreover, they also showed that a relatively small amount of AMOTp80 (5%) to that of AMOTp130 was enough to induce a migratory phenotype, indicating that AMOTp80-induced cell migration function is dominant over AMOTp130 in endothelial cells [13]. In our study, we found that the two isoforms were co-expressed in PCa cells with similar levels but possessed distinct functions *in vitro*. Our studies showed that overexpression of AMOTp80 in PC3-mm2 cells increased cell proliferation, while overexpression of AMOTp130 alone did not. Co-expression of AMOTp80 and p130 led to a phenotype similar to that observed with AMOTp80 alone, although the effect of AMOTp80 was slightly attenuated. Thus, our observation in PCa cells is distinct from that reported in aortic endothelial cells.

It is possible that AMOT isoforms exhibit different functions through differential regulation of the Hippo pathway. AMOTp130 was previously shown to interact with YAP directly through the AMOTp130 PPXY motif, which induces YAP cytoplasmic retention and degradation, resulting in inhibition of YAP nuclear translocation [17, 22, 23]. In contrast, our study showed that AMOTp80 increases YAP nuclear translocation through modulating the upstream components of the Hippo pathway. Several studies have shown that AMOTp80 could interact with Merlin (a tumor suppressor) to regulate the Hippo pathway and this signaling pathway has been shown to regulate human meningioma cell growth [18, 24–27]. Recently, the predicted coiled-coil (CC) domains of AMOTp80 and AMOTp130 were shown to interact with the helical region between the FERM domain and the inhibitory CTD of Merlin [5]. Thus, it is likely that AMOTp80/Merlin binding prevents Merlin to activate the Hippo pathway in PCa.

AMOTp80 was also reported to increase proliferation through other pathways. Studies by Ranahan et al. [6] showed that AMOTp80 enhances the proliferation of breast cancer cell line MCF7 through increasing ERK1/2-phosphorylation. Another study showed that AMOTp80 promotes ERK1/2 activity through Rac1 GTPase [5]. Other studies showed that AMOTp80 affects ERK1/2 activity through YAP-dependent transcriptional regulation of amphiregulin, a growth-promoting epidermal growth factor receptor ligand, which leads to ERK1/2 activation [32, 33]. We have also examined whether AMOTp80 had an effect on ERK1/2 phosphorylation and found that overexpression of AMOTp80 in PC3-mm2 did not increase ERK1/2 phosphorylation either under normal growth condition or under serum starvation followed by serum stimulation condition (data not shown). Similarly, knockdown of AMOTp80 in C4-2B4 cells did not decrease ERK1/2 phosphorylation (data not shown). These observations suggested that AMOTp80-mediated PCa cell proliferation is not mediated through ERK1/2 activation.

Our observation that AMOT expression modulates PCa cell proliferation through the Hippo pathway raises an interesting question concerning the role of Hippo pathway in prostate organogenesis and PCa progression. Hippo pathway was originally identified in Drosophila as a signaling pathway that regulates organ size [34–36]. Lu et al. [37] showed that Hippo signaling is also a potent *in vivo* growth and tumor suppressor pathway in the mammalian liver. Increase in the nuclear localization of YAP has been shown in liver and PCa and down-regulation of LATS1/2 expression is observed in metastatic prostate cancer [38]. Our finding that AMOTp80 expression increased YAP nuclear localization provided one of the upstream regulators for the inhibition of the Hippo pathway in PCa.

In this study, we showed that the levels of AMOT are higher in LNCaP and its subline C4-2B4 cells, and AMOT are relatively low in PC3-mm2 and DU145 cells. PC3-mm2 and DU145 cells were derived from bone metastasis and brain metastasis, respectively, of PCa patients. As AMOT is a polarity protein involved in tight junction formation [3], it is possible that low levels of AMOT in PC3-mm2 and DU145 cells may reflect a loss of cell polarity during PCa progression. Similarly, AMOTp130 and p80 levels were found to be low or undetectable in the highly metastatic breast cancer cell line MDA-MB231 cells [6]. Ortiz et al. [39] have previously shown that AMOTp80-Cadherin 11 interaction is involved in promoting cell migration, rather than cell proliferation, in PCa cells. Because AMOTp80 can interact with many proteins, including Cadherin-11, AMOTp80 overexpression or knockdown may affect the interactions of AMOTp80 with other cellular proteins. Given that AMOTs are adaptor proteins that interact with many proteins besides those in the Hippo pathway, the roles of AMOTs in cells may be dependent on both the levels of the AMOT isoforms and the cellular context. The possibility that the two AMOT isoforms may work against each other may explain in part why we did not see a significant correlation between AMOT expression and patient outcome, metastasis versus primary tumors, or copy number alteration using several PCa patient datasets (data not shown). Thus, how AMOTp80 contributes to PCa tumor progression requires further assessment.

In conclusion, we have shown that AMOTp80 plays a role in PCa cell proliferation by signaling through the Hippo pathway. Future investigation of whether AMOTp80-MST1-LATS-YAP-BMP4 signaling pathway is involved in various stages of PCa progression is warranted.

## MATERIALS AND METHODS

### Cell lines and antibodies

C4-2B4, DU145, LNCaP, PC3, HEK293, 293FT, and Phoenix cells were from American Type Culture Collection. PC3-mm2 was kindly provided by Dr. Isaiah Fidler (University of Texas, M. D. Anderson Cancer Center). All the cell lines were authenticated by short tandem repeat DNA profiling. Anti-YAP, anti-phospho-YAP (Ser127), anti-LATS1, anti-phospho-LATS1 (Thr1079), anti-MST1, anti-MST2, anti-ERK1/2, anti-phospho-ERK1/2 (Thr202/Tyr204), and anti-lamin A/C were from Cell Signaling Technology (Boston, MA).

### RNA isolation and real-time RT-PCR

Total RNA was isolated using RNeasy Mini Kit (Qiagen) and then was reverse transcribed with Reverse Transcription Kit (Applied Biosystems). The resulting cDNA was used for real-time PCR by using SYBR green reagent (Applied Biosystems). Data were normalized to GAPDH as an endogenous control. Nucleotide sequences of primers used for real-time PCR were listed in Table 1.

**Table 1:** Designed PCR primers sequences

Oligo Name	Genebank No.	Primer sequences(5′→3′)
AMOTp130**(Full length)**	NM_001113490.1	**F:** GGATCCATGAGAAATTCTGAAGAACAGCCAA (**BamHI**)**R:** GCGGCCGCTTAATGATGATGATGATGATGATGGATGAGATATTCCACCATCTCTGC (**NotI**)
AMOTp80**(Full length)**	NM_133265.2	**F:** GGATCCGCTATGCCTCGGGCTCAGCCATCCTCT (**BamHI**)**R:** GCGGCCGCTTAATGATGATGATGATGATGATGGATGAGATATTCCACCATCTCTGC (**NotI**)
AMOTp130**(Q-PCR)**	NM_001113490.1	**F:**CGTTTGCTACAAGAGCAGCTT**R:**GTTCTTGTCGAGCAGCATGAG
AMOTp80**(Q-PCR)**	NM_133265.2	**F:**GCTGCCTAAACTGGTAGACCC**R:**CTGACTGGAGGTGGTGGTAGT
AMOTp80-F2		**F:**CTCGCAGCTCTTTGCAAAAA
AMOTp80-F3		**F:**CAATAAGCGTTGCCTTGACA
AMOTp80-F4		**F:**ACTGCCGCTGCTACTGCTGC
FOXM1	NM_001243089.1	**F:** CGCAGCATCAAGCAAGAG**R:** CCATGTAAGAGTAGGGTGGC
CCND1	NM_053056.2	**F:** TCGTCACCCTTCTCCACTT**R:** CTTGGCGCAGACCTTACA
GAPDH	NM_002046.4	**F:**TGATGACATCAAGAAGGTGGTGAAG**R:**TCCTTGGAGGCCATGTGGGCCAT

### Generation of AMOT antibodies

Purified GST-AMOTp80 was used as immunogen for antibody generation. GST-AMOTp80 protein was expressed and purified as follows. cDNA for AMOTp80 was generated by PCR using primers Amot-F1 and Amot-R1 (Table 1) and pCR4-TOPO-AMOTp80 as template. The PCR product was ligated into pCR2.1-TOPO vector and the DNA sequence confirmed by using oligo Amot-F2 to F4 (Table 1). The AMOTp80 insert was removed from pCR2.1-AMOTp80 by digesting with BamHI and NotI restriction enzymes and subcloned into pGEX-4T1 or pET-28b vectors to express AMOTp80 as GST-tagged or 7xHis-tagged proteins, respectively. The GST-AMOTp80 protein was expressed in E. coli and purified through GST-agarose affinity matrix. The purified GST-AMOT-his7 fusion protein was used to immunize rabbits for the development of polyclonal anti-AMOT antibodies.

Polyclonal anti-AMOT antibody was further purified as follows. AMOT-his7 was expressed in E. coli and purified through Ni-NTA agarose. Purified AMOT-his7 was resolved on SDS-PAGE and transferred onto nitrocellulose membrane or directly spotted onto nitrocellulose membrane. AMOT-his7 protein that was immobilized on the membrane was then used as affinity matrix to bind anti-Amot antibodies. Antibodies were eluted from the nitrocellulose membrane using Gentle Elute (BioRad).

### Construction of AMOTp80 and AMOTp130 retroviral vector

Full length cDNAs of human AMOTp130 (NM_001113490.1) and AMOTp80 (NM_133265.2), with 7-histidine tag at C-termini, were generated by PCR using AMOT full length primers (Table 1) and subcloned into retroviral vectors pBMN-I-GFP and pBMN-I-NEO, respectively. The resulting plasmids were named pBMN-AMOTp130-GFP and pBMN-AMOTp80-NEO, for AMOTp130 and AMOTp80, respectively.

### Generation of PC3-mm2 cells overexpressed AMOTp80 or AMOTp130

To stably overexpress AMOTp130 or AMOTp80 in PC3-mm2, bicistronic retroviral vector containing AMOTp130 or AMOTp80 with 7-histidine tag at their C-termini were used to infect PC3-mm2 cells. Cell culture medium containing retroviral particles were generated by transfecting Phoenix packaging cells with pBMN-AMOTp130-GFP and pBMN-AMOTp80-Neo using PEI as described [40]. Retroviruses were also generated from pBMN-I-GFP or pBMN-I-Neo vectors and used as controls. At 48hr post-transfection, the cell culture supernatants were harvested, filtered with 0.45um filter, and used to infect PC3-mm2 cells with 8μg/ml polybrene. PC3-mm2 cells expressed AMOTp130 or AMOTp80 were selected by FACS sorting (for GFP) or G418 (for Neo) selection, respectively. PC3-mm2 cells that co-expressed both AMOTp130 and AMOTp80 were selected by both G418 and FACS.

### Western blot

Cells were pre-treated with 2mM sodium vanadate for 1 hour and scraped with lysis buffer, which included 1% Triton X-100, 50mM Tris HCl (pH 7.2), 150mM NaCl, protease inhibitor cocktail (Sigma), 1mM sodium vanadate, and 50mM NaF. After centrifugation (13000rpm/min, at 4°C) for 30min, supernatant were collected and used for western blot. Total proteins were resolved by 4-12% gradient NuPage gels (Invitrogen) and electroblotted onto nitrocellulose membrane. The membranes were blotted with antibody and antibody binding was detected by chemiluminescence (Pierce). Western blot with anti-Actin antibody (Santa Cruz) was used as a control.

### Generation of subcutaneous PCa tumors

The luciferase-expressing C4-2B4 or PC3-mm2 cells were injected under the skin of male SCID mice. Tumor growth was monitored using bioluminescence imaging. All manipulations were approved under the MD Anderson Cancer Center Institutional Animal Care and Use Committee.

### Immunohistochemistry

Sections (4μm) were immunostained with affinity-purified anti-AMOT antibody. Endogenous peroxidase activity was blocked with 3% hydrogen peroxide (H_2_O_2_) for 20min at room temperature. Antigen retrieval was done by microwave in citrate buffer (pH 6.0) for 15min. After blocked by normal goat serum at 37°C for 30min, sections were incubated with affinity-purified anti-Amot antibody at 4°C overnight. Antibody binding was detected by using VECTASTAIN ABC kit (Vector Laboratories, Burlingame, CA) according to the manufacturer's instructions. Controls were immunostained similarly but without the primary antibody.

### Cell proliferation assays

Cells were plated into 6-well plates (80,000 cells/well) in duplicate. At the indicated times, the cells were removed from the plates by trypsin digestion and the cell numbers counted with a hemocytometer.

### Cell migration assay

Cells were starved overnight in serum free RPMI 1640. Cells (1×10^5^) in 300ul serum-free medium were seeded into a 24-well insert (BD Falcon) and placed into chambers containing medium with 1% FBS. After incubation at 37°C for 8hr, cells were stained with 1 mM Calcein AM (Invitrogen) and the number of cells that had reached the other side of the filter was counted under a fluorescence microscope.

### Generation of C4-2B4 cells with *Amot* knockdown

To establish AMOT shRNA knockdown C4-2B4 cell lines, three shAMOT (shAMOT#1,2,3) in pGIPZ lentiviral vector (Addgene, MA) were screened and the cells infected with lentivirus, which expressed shAMOT with two best knockdown vectors (shAMOT#1 and shAMOT#2), were used for functional studies. Control C4-2B4 cells were infected with pGIPZ lentiviral vector.

### Immunofluorescence

Cells were plated in 24-well plate with coverslips and allowed to grow and adhere overnight. Cells were fixed with 100% cold Methanol at −20°C for 10min. Non-specific activities were blocked with buffer containing 1% BSA, 0.5% Tween 20 and 10% serum homologous with secondary antibody for 1 hour, and then cells were incubated with anti-AMOT antibody overnight at 4°C. After washed, cells were incubated for 45min in darkness with FITC488-conjugated donkey anti-rabbit antibody (1:500) (Jackson Immnuoresearch). After cells were stained with DAPI (1:500, Molecular Probes), the slides were mounted with mounting media from Vector Laboratories, viewed on microscope.

### Nuclear cytoplasmic fractionation

Nuclear and cytoplasmic protein fractions were prepared by using NE-PER reagents (Pierce). Lamin A/C and NudC were used as nuclear and cytoplasmic marker, respectively [41].

### Effect of LDN193189 on cell proliferation

PC3-mm2 and C4-2B4 cells were treated with BMPR inhibitor LDN193189 (Axon Medchem, Netherland) with final concentrations of 50 nM, 100 nM, 200 nM, and 500 nM, respectively. Cells were seeded in 6-well plates in duplicate and incubated with AMOTp80/p130 or shAMOT, and then they were treated with different concentrations of LDN193189 for 48 hrs. Viable cells were counted by hemocytometer.

### Statistical analysis

The SPSS 19.0 software was used for statistical analysis. t test was used for the comparisons between groups.

## Abbreviations

AMOT: Angiomotin
NF2: neurofibromatosis type 2
PCa: prostate cancer
